# Reduction of simultaneous ordering rate of high sensitivity C-reactive protein and erythrocyte sedimentation rate in a veteran affairs healthcare system

**DOI:** 10.3389/frhs.2025.1729117

**Published:** 2026-04-22

**Authors:** Luke Valencia

**Affiliations:** 1 University of Kansas Medical Center, Kansas, KS, United States; 2 Laboratory Medicine, Department of Veteran Affairs, Sacramento, CA, United States

**Keywords:** C-reactive protein (CRP), cost saving, erythrocyte sediment rate, ESR, hs-CRP, lean, veteran affairs

## Abstract

It is widely recognized that the ESR (Erythrocyte Sedimentation Rate) and hs-CRP (C-Reactive Protein) are comparable inflammatory markers. In this reported veteran population, there is a significant correlation between the two assays. The simultaneous ordering of ESR and hs-CRP for evaluation of acute inflammation is unnecessary and wasteful. Various hospitals have implemented processes to reduce co-ordering habits through best practice advisories (BPAs) and/or educational interventions. In this retrospective study spanning twelve months, the Northern California Veteran Affairs Hospital System had a co-ordering rate of approximately 72% (number of hs-CRP orders with a co-ordered ESR compared to the total number of hs-CRP orders). After implementing a best practice ordering advisory and providing education to the clinical teams, there was a significant decrease in co-ordering of the assays (−34%, *p*-value = 0.00036). There was also a significant decrease in standalone ESR ordering and standalone hs-CRP ordering (*p*-value = 0.0003 and 0.0042, respectively). Departments receiving both interventions, an ordering advisory and education, had the largest decrease in co-ordering habits, the largest decrease seen being more than 56%. Proper implementation of ordering advisories paired with education results in better test utilization and lower co-ordering rates.

## Introduction

Human inflammatory conditions can be assessed by various blood biomarkers. Although non-specific, the most common biomarkers used in the clinical laboratory are the C-reactive protein (hs-CRP) and erythrocyte sedimentation rate (ESR) (1). C-reactive protein is produced by liver hepatocytes in response to acute inflammation (2). As such, an elevated measurement serves as a direct indicator of an acute inflammatory process. Its relatively short half-life (19 h) makes it especially suitable for assessing rapid and acute clinical changes. ESR is an older and less direct measurement of inflammation, which has historically been used as an “sickness index.” (3) It is affected by many factors, such as fibrinogen level, anemia, and other hematological abnormalities (4). Although the ESR test is better suited for some chronic inflammatory conditions, measurement of hs-CRP is the preferred method of detection for acute inflammation (5). In most acute clinical conditions, the two assays are strongly correlated. In these cases, there is little to no added benefit to using them together (6). In fact, best practice recommendations from the *Choosing Wisely Campaign* strongly advise against co-ordering of ESR and hs-CRP for acute inflammatory conditions (7). The implementation of this recommendation has been shown to lead to less medical waste, better use of phlebotomy and technologist time, and a reduction of associated costs (8). As a result, institutions have begun implementing methods to reduce co-ordering habits of the two tests. Within these institutions, ordering advisories and education have been used to increase awareness and encourage evidence-based ordering practices of hs-CRP and ESR assays.

This quality improvement project had two aims. The first was to establish the correlation between hs-CRP and ESR measurements within a veteran population at a VA medical center. This part of study was implemented through a 12-month retrospective review of all hs-CRP and ESR assays which were ordered within six hours of each other. These hs-CRP and ESR values were compared for correlation and concordance. The project's second aim was to implement best practice recommendations to reduce co-ordering of the assays. This was achieved through an ordering advisory and educational interventions. The outcome was measured through changes in ESR and hs-CRP co-ordering practices. Here I report the implementation of ordering advisories, paired with education, results in better test utilization and lower co-ordering rates of hs-CRP and ESR.

### Literature review

#### Inflammation overview

Inflammation is the body’s response to tissue damage arising from wounds and infections. When cells become damaged, they release damage-associated molecular patterns (DAMPs). These DAMPs include molecules like DNA, histones, adenosine triphosphate (ATP), and various interleukins. DAMPS interact with the innate immune system to recruit neutrophils, monocytes/macrophages, and natural killer cells to the affected areas through chemo-attractants that recruit the cells via their cell surface pattern receptors (9). The main role of these white blood cells is to phagocytize dying tissue and foreign material. DAMPs act on the surrounding epithelial cells and immune cells to induce cytokine release. Cytokines, particularly interleukin-6 (IL-6), induce a systemic response acting on the liver to release acute phase proteins, such as C-reactive protein (hs-CRP), alpha1-antitrpsin and haptoglobin, throughout the body. During acute inflammation there is a rapid release of acute phase proteins within hours of tissue damage. The release of cytokines and proteins facilitates the degradation of dying cells and the destruction of microorganisms. These also assist in the further recruitment of macrophages and other specialized cells to the area of tissue damage. During cases of infection, additional pattern-recognition receptors (RPRs), known as pathogen-associated molecular patterns, work alongside DAMPs to amplify the inflammatory pathway. These include Toll-like receptors, retinoic acid-inducible gene I-like receptors and C-type lectin receptors. These RPRs mediate nuclear factor kappa-B (NF-*κ*B) and JAK-STAT intracellular pathways (10). The intracellular pathways further upregulate the inflammatory process. For instance, these pathways may induce an attack against an extracellular pathogen or incite cellular apoptosis in the event of an intracellular pathogen.

After resolution of the injury, there is a release of self-associated molecular patterns (SAMPs) and specialized pro-resolving mediators (SPMs). These molecules downregulate the immune response through inhibition of DAMPs (11). Without resolution of injury, the body may enter a state of chronic inflammation. During chronic inflammation states, hepatic secretion of acute phase proteins will decrease, but detectable amounts of these proteins remain present within the body. This phenomenon is seen in autoimmune conditions, such as systemic lupus erythematosus (SLE) and rheumatoid arthritis (RA). Resolution of inflammation may be inhibited by either continued presence of inflammatory stimuli or an abnormal white blood cell response to pro and anti-inflammatory molecules. Continued infiltration of white blood cells may eventually lead to permanent tissue damage from prolonged exposure to the cells' byproducts, which include proteases and reactive oxygen species (12). Over time, persistent cellular damage from activation of the inflammatory process can lead to chronic health conditions.

#### Inflammation markers

The inflammation pathway is facilitated by cell surface pattern receptors, recruitment of inflammatory cells, and release of inflammatory biomarkers (13). Inflammatory biomarkers are the cytokines and proteins that guide the inflammatory process. In the clinical setting, these are used to assess the disease process and severity of inflammation. Well-known clinical inflammatory biomarkers are TNF-alpha, IFN-gamma, IL-1, IL-6, IL-8, IL-10, IL-12, and multiple acute phase proteins: C-reactive protein (hs-CRP), fibrinogen, ferritin, and serum amyloid A (14). ESR serves an indirect measurement of inflammation, with its measurement determined predominantly by fibrinogen protein levels (15). Although many biomarkers have been studied, hospitals do not often offer an abundance of biomarker assays on their test menus. hs-CRP and Erythrocyte Sedimentation Rate (ESR) are commonly available biomarkers.

#### C-reactive protein (hs-CRP) as an inflammation marker

**Figure 1:**
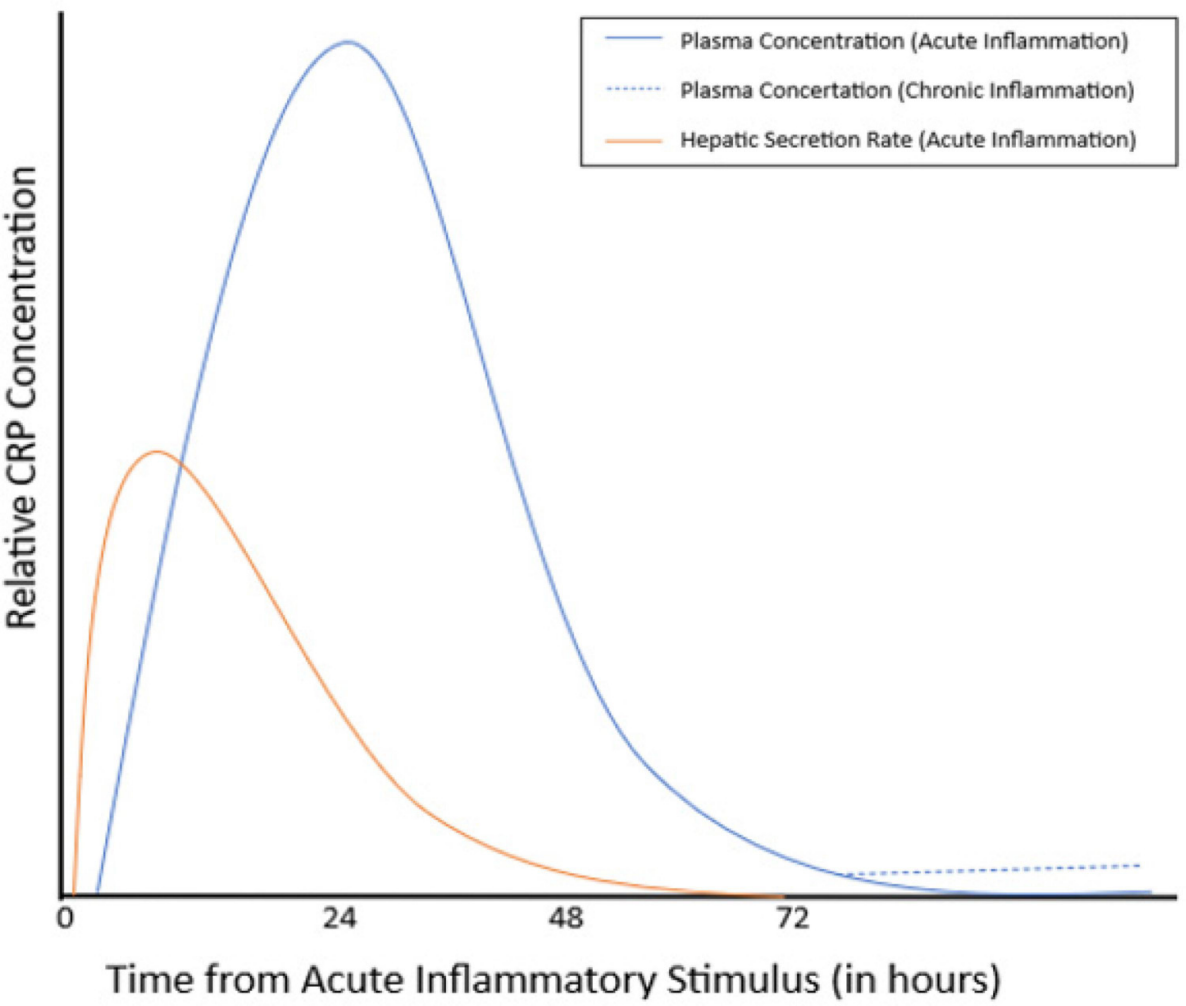
hs-CRP levels in response to acute and chronic inflammation adapted from chen L et al. (10).

hs-CRP is an acute phase reactant which directly correlates with levels of inflammation in the body. hs-CRP is regulated by immune cells through means of IL-6 and interferon-alpha release of inflammasomes. IL-6 stimulates hepatocytes to synthesize and release hs-CRP, leading to a rise in detectable serum hs-CRP levels. The main role of hs-CRP during the inflammatory process is activation of the complement pathway through the C1q molecule and Fc receptors of IgG (16). This activation helps to promote opsonization of pathogens and release of other pro-inflammatory molecules in response to cellular damage. It also creates a positive feedback loop, which continues to stimulate the inflammatory response. After resolution of the injury, interferon-alpha works on hepatic cells to inhibit production of hs-CRP. When cellular damage or bacterial exposure occurs, hs-CRP levels may begin to rise within six hours. The rise can be upwards of 100–1,000-fold and often returns to baseline within a few days following resolution of the condition (8). In chronic states of inflammation, low levels of detectable hs-CRP are present in the body until remission (Figure 1).

hs-CRP is an annular pentameric molecule made up of five identical 23kD globular subunits (17). Each subunit has a calcium-regulated binding site specific to phosphocholine in the lipopolysaccharides and phospholipids of bacteria. hs-CRP functions as a part of the innate immune system to recognize some species of gram-positive bacteria and ligands expressed on damaged or dead host cells (16). The binding of hs-CRP causes an upregulation of the complement system through activation of the classic complement pathway via C1q binding and furthers the inflammatory cascade through stimulating the release of IL-10.

**Figure 2:**
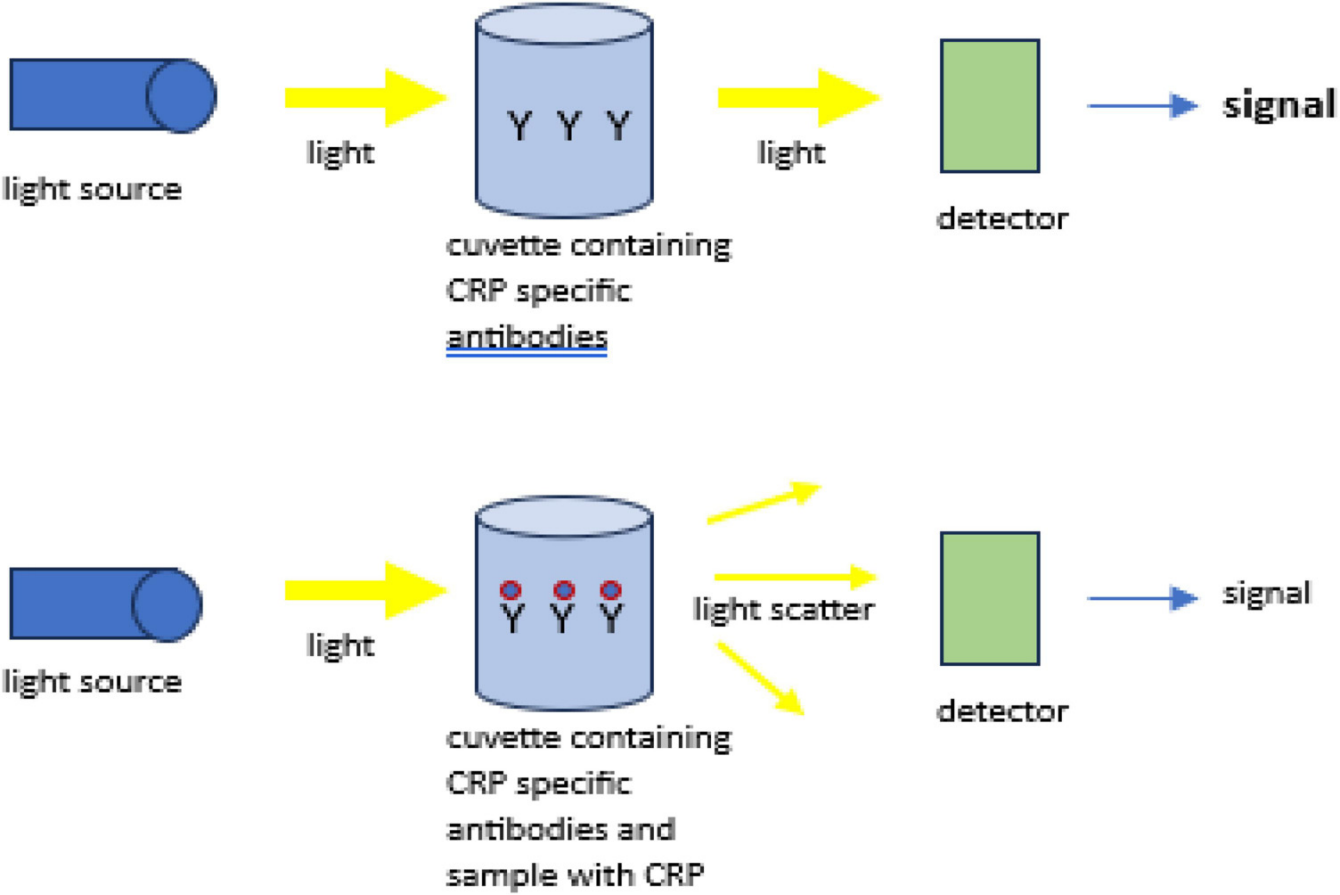
Turbidimetric immunoassay of hs-CRP measurement, adapted from pohanka et al. (13).

There are two quantitative assays for hs-CRP on the market. The two assays available for clinical utility are the standard hs-CRP and the high-sensitivity hs-CRP (hs-CRP). Both assays measure the same hs-CRP biomarker using a similar methodology, with the only difference in the two assays being their linearity. The hs-CRP has a lower limit of linearity, detecting concentrations below the cutoff threshold of the standard hs-CRP. Standard hs-CRP measures serum concentrations at and above 10 mg/L, while hs-CRP detects levels as low as 1 mg/L. This makes hs-CRP useful for risk stratification of atherosclerosis-associated coronary artery disease (18). When measuring levels above 10 mg/L, the two assays are virtually identical in terms of performance. The most common method to detect hs-CRP is an automated turbidimetric immunoassay, which measures absorbance at 340 nanometers (19). This method utilizes hs-CRP-specific antibodies that bind to the molecules to form a large, insoluble complex. The insoluble complex scatters light, which is measured by a detector (Figure 2).

The delta in the absorbance, defined as the difference in absorption before and after the light is scattered, correlates to the concentration of hs-CRP in the patient's serum (13).

Aside from cardiac care, hs-CRP is most useful for detecting acute inflammation in the clinical setting (9). People within the general population have a serum hs-CRP concentration of less than 10 mg/L. When hs-CRP concentrations rise above this level, it may be indicative of an inflammatory process. This inflammation may be due to cellular damage or infection. Diet has also been shown to play a minor role in serum hs-CRP concentration (20). The sensitivity of hs-CRP for inflammation is upwards of 80%, and it is greater than 90% in certain conditions (21). In cases of infection, particularly sepsis, hs-CRP becomes significantly elevated. This elevation surpasses levels that would be expected with non-infectious tissue damage. The degree of increase in hs-CRP serum levels is useful for distinguishing infectious inflammation from inflammation due to non-infectious conditions (22). Non-infectious conditions which cause tissue damage include shear trauma from surgery, autoimmune conditions such as RA or SLE, and malignancy (23).

Although hs-CRP is a sensitive test, it faces limitations. Mild elevations of hs-CRP, values at or around 10 mg/L, suggest an inflammatory disease process. However, this may not always be the case. Noninflammatory processes can contribute to the elevated, yet relatively low, levels of hs-CRP. These conditions include smoking, obesity, insomnia, depression, diabetes, and diet (24). Chronic inflammation may also lead to an elevated baseline hs-CRP (25). Elevated baseline hs-CRP levels can make it difficult to determine whether an acute disease is developing. Another limitation hs-CRP faces is one shared by all turbidimetric chemistry assays. Improper collection or interfering substances, such as lipemia or hemolysis, can cause erroneous results.

#### Erythrocyte sedimentation rate (ESR)

**Figure 3:**
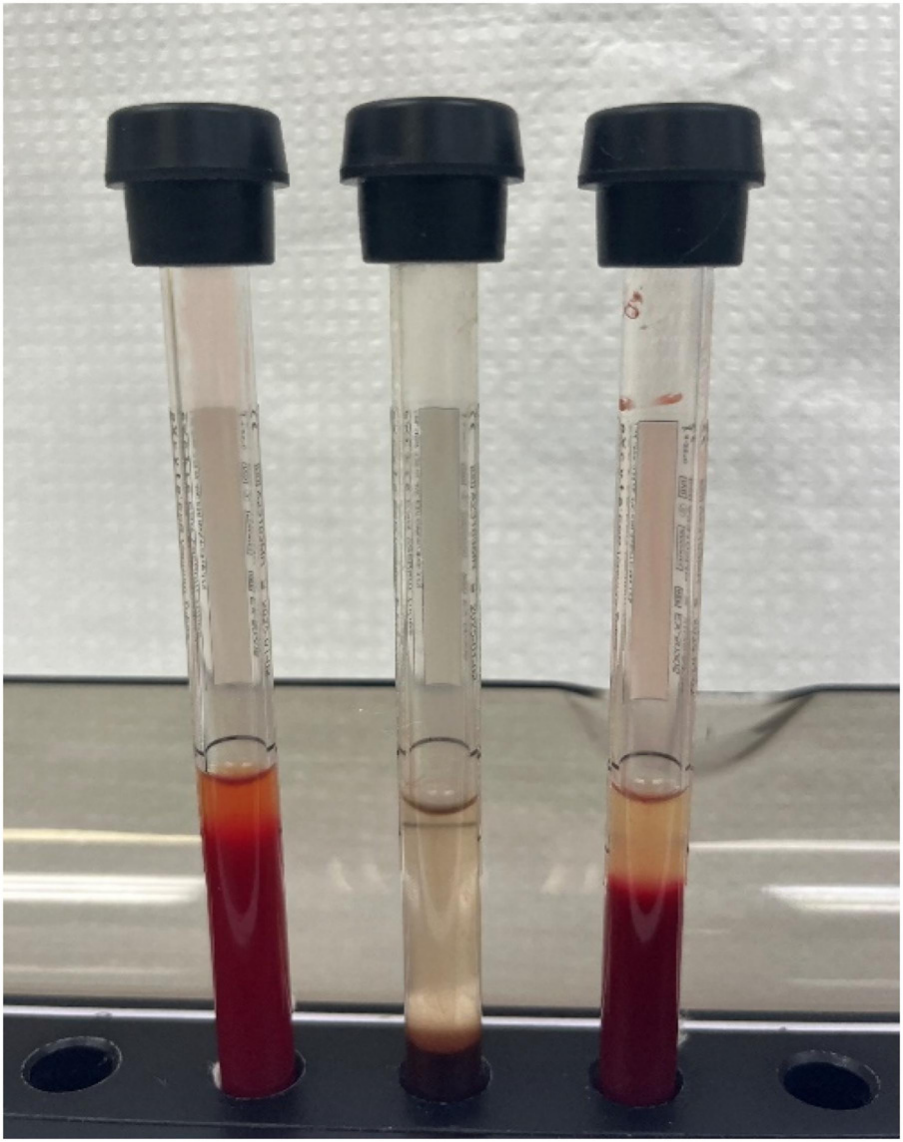
Whole blood at rest showing different separations of erythrocytes from plasma due to variability among samples, photographed by Valencia at Mather VA Hospital.

Compared with hs-CRP, ESR is a different approach to assessing inflammation in the body. It is an indirect measurement of red blood cell sedimentation rate using the properties of fibrin, acute phase reactants, and other factors associated with inflammation. The assay was developed in the 1920s and is one of the most widely used non-specific indicators of inflammation in the clinical laboratory (26). The principles of the methodology have not changed much since its implementation. When whole blood is at rest, the erythrocytes begin to aggregate and separate from plasma (18) (Figure 3).

**Figure 4:**
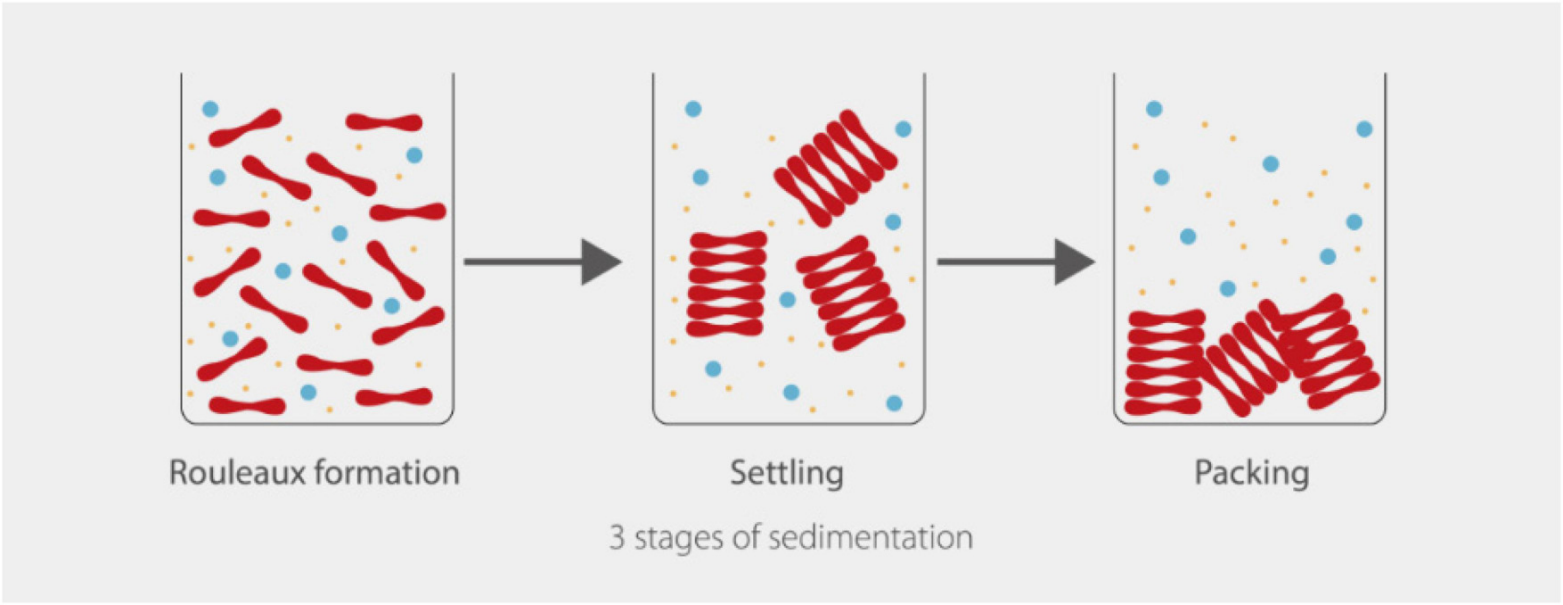
Three stages of sedimentation, adapted from mindray (29).

Inflammatory proteins in the serum, predominantly fibrinogen, promote red cell interactions and lead to a more rapid dispersion (27, 28). At the cellular level, reversible two and three-dimensional rouleaux structures form. At the macroscopic level, red cell aggregation appears as serum separating out from the cellular components (29) (Figure 4).

When placed inside a vertical tube, the separation can be quantitatively measured in millimeters per hour. Individuals with elevated acute phase reactants will have an abnormally increased ESR. More severe inflammation leads to higher quantities of acute phase reactants in the blood, which correlates with higher ESR values.

Current methods build off the principles of erythrocyte aggregation. As was done historically, ESR is reported in millimeters per hour. This test is still performed manually in many facilities. Some facilities use instrumentation to reduce turnaround time. The manual method takes up to an hour, whereas newer instrumentation has reduced this time to less than a minute (30). The instrumentation may use the properties of centrifugation to speed up red cell sedimentation, microscopy to observe how rapid the aggregation occurs, and computerized recording of the number of millimeters per hour through light transmission or red cell reflectance. Interfacing these instruments with the laboratory informatics system is also possible. This allows for automated validation, further reducing the time it takes to obtain a result.

When utilizing ESR in the clinical setting, it is most useful for detecting chronic inflammation (31). This is due to the long half-life associated with ESR. ESR may take upward to 24 h before becoming elevated from acute inflammation and may take one week to decrease after resolution of the inflammatory condition. The general population has a normal ESR value of less than 20 mm/hr; however, it is important to correct the upper limit of normal for age. The upper limit of normal is the patient's age divided by two (13). For example, the upper limit of normal for an 80-year-old male would be (80/2) = 40 mm/hr. When ESR values rise above this corrected cutoff of normal, it is indicative of an inflammatory process.

Certain conditions may impact the reliability of ESR and limit its utility in the clinical setting. There are various conditions that lead to an increase in ESR. The most common factor contributing to erroneous ESR results is anemia. Anemia changes the ratio of erythrocytes to plasma, increasing the aggregation rate of red blood cells. This can be a major concern for false positive results, as up to 41% of patients admitted to the hospital have some form of anemia (32). Myeloma proteins, cold agglutinins, and macro-globulins, although uncommon, are additional factors that cause an increase in the ESR that is not attributed to inflammation (33, 34). Renal failure and pregnancy, although not inflammatory processes, lead to an increase in fibrinogen causing an increase in ESR values (22). When ruling out inflammation in patients with end stage renal disease or pregnancy, alternative biomarkers should be considered.

In addition, several technical errors can contribute to notable changes in ESR values. Use of heparin-based blood collection tubes, rather than EDTA or sodium citrate, is associated with false ESR results. Small deviations in the test setup can have large consequences on the results, too. For example, a 3-degree change in the vertical tube method leads to a 30% increase in the ESR.

Many conditions and factors may falsely decrease ESR values (35).

Polycythemia is an increase in red blood cell numbers. Similar to anemia, the change in the erythrocyte to plasma ratio alters the red blood cell interactions. The increase in red cells results in falsely lower ESR. Abnormal red blood cell morphologies also impact red blood cell aggregation. At the microscopic level, abnormal morphologies impede rouleaux formation, thus decreasing the ESR. An increase in white blood cells also impairs red cell aggregation. Hypofibrinogenemia, dysfibrinogenemia, and afibrinogenemia are conditions where the level of fibrinogen is abnormal or non-existent. Being a primary acute phase reactant influencing the ESR, fibrinogen level decreases will lead to lower readings of ESR. Increases in serum bile salt concentrations and medications, like low molecular weight dextran and valproic acid, also decrease ESR. Technical errors notable for decreasing ESR include samples not being at room temperature and performing the test longer than two hours after collection (4).

#### Application of hs-CRP and ESR

hs-CRP’s rapid response to inflammatory stimuli, as well its rapid resolution after cessation of stimulus, makes the biomarker useful in clinical decisions associated with acute inflammation. hs-CRP is useful for both infectious and non-infectious causes, with the severity of the rise of hs-CRP levels being helpful in distinguishing between the two. In most causes of acute inflammation, hs-CRP levels parallel the patient's course with onset and resolution. hs-CRP levels also tend to increase in cases of chronic inflammation. However, the correlation is not as strong between the hs-CRP levels and severity of the disease state (36). Autoimmune conditions, particularly SLE, have suppressed hs-CRP secretion with inflammation. This is due to type I interferons expressed in SLE patients. The interferons act on the liver cells to reduce hs-CRP secretion, leading to reduced serum concentrations. Another example of the hs-CRP's limitation in assessing chronic inflammation is RA. A study demonstrated that approximately 60% of patients show no increase in acute phase reactants, including hs-CRP (37). Certain chronic conditions may require additional testing or more specific biomarkers alongside the hs-CRP to fully assess the patient's condition and track the resolution or severity of the disease.

The ESR, as an indirect method of inflammation detection, incorporates a range of acute phase reactants involved in the inflammatory process with long half-lives. Because of this, it has a slower response to changes in inflammatory stimuli and resolution of disease. Notable changes in ESR results may take up to a week, making it difficult to use for acute conditions. It is particularly useful in assessing chronic inflammation. An ESR can be used to follow inflammatory patterns over months. An example of a condition where using the ESR may be beneficial is in patients with SLE, where hs-CRP is unable to consistently measure inflammation. Since the ESR's measurement is influenced by other acute phase reactants and is not impacted by type I interferons, it can more accurately reflect the patient's condition (38). The ESR also has a correlation between its elevation and the patient's resolution of symptoms (39). Similarly to the hs-CRP, more severe increases in the ESR may be used to distinguish between infectious and non-infectious etiology. ESR levels over 100 mm per hour were commonly associated with infection (40). Another indication for ESR utilization is for rule out in preliminary cases of chronic osteomyelitis. One study demonstrated that an ESR of greater than 60 mm per hour in patients suspected of bone infection is suggestive of osteomyelitis and had a sensitivity of 74%. However, it had a relatively low specificity of 56%. Algorithms utilizing the ESR for osteomyelitis require an additional biomarker, such as hs-CRP, to help distinguish osteomyelitis from soft tissue infections. Implementing additional testing, such as wound culture, is also required for a proper diagnosis and treatment plan. Using ESR in other chronic conditions can prove difficult, as the previously mentioned limitations, including anemia, are common in these patient populations, making it difficult to distinguish between true values and false elevations.

Literatures have documented a few cases where hs-CRP and ESR are discordant. As mentioned, patients with SLE may not have an elevated hs-CRP, thus requiring an ESR or comparable biomarker to accurately follow their clinical course (41). In cases of RA, neither inflammatory marker may capture the patient's condition. These cases may require more specific markers, like rheumatoid factor, to assess the patient's inflammation. In cases of osteomyelitis, both biomarkers and additional testing may need to be utilized to fully understand the source of inflammation. In other settings of inflammation, the two assays perform similarly and moderately correlate (2). In other autoimmune conditions, where traditionally the ESR was the primary biomarker used, such as polymyalgia rheumatica and giant cell arteritis, studies have demonstrated concordant results between the ESR and hs-CRP. The studies have shown that hs-CRP is almost always elevated, in around 99% of patients, whereas the ESR may have a false negative rate in up to 20% of cases (42).

#### Best practice recommendations

ESR relies predominantly on fibrinogen as the acute phase protein that impacts its result. Fibrinogen has a half-life of about 40 h. This causes the half-life of the ESR to be vastly longer than that of the hs-CRP. Thus, with ESR being a cumulative measurement of inflammation, it is better for the management of chronic conditions. In these cases, it is adequate to only check the marker once every couple of months, especially in conditions that may not be well-suited for hs-CRP testing. hs-CRP testing is better suited for acute conditions where there is an immediate onset. The rapid changes in acute conditions need to be reflected by a biomarker with a short half-life and quickly elevated in response to new stimuli (31). hs-CRP is also less affected by other conditions and has fewer limitations than that of the ESR. Anemia and age are frequent interferences with ESR, particularly age in the veteran population, making hs-CRP a superior test.

One notable recommendation that incorporates these principles comes as a joint effort from the American Society of Clinical Pathologists (ASCP) and the American Board of Internal Medicine (ABIM). Their recommendation was released in 2015 and published in 2020 under the *Choosing Wisely Campaign: Thirty-Five Things Physicians and Patients Should Question*. The recommendation states: “Do not order and erythrocyte sedimentation rate to look for inflammation in patients with undiagnosed conditions. Order a C-reactive protein to detect acute phase inflammation.” The reasoning for hs-CRP test preferred over ESR is “hs-CRP is a more sensitive and specific reflection of the acute phase inflammation than the ESR. In the first 24 h of the disease process, hs-CRP will be elevated, while ESR may be normal. If the source of inflammation is removed, hs-CRP will return to normal within a day or so, while ESR will remain elevated for several days until excess fibrinogen is removed from the serum.” (43) The goal of this recommendation is to help guide practitioners to provide a higher quality of care using evidence-based testing and help reduce cost through effective utilization of resources and medical professionals' time.

In addition to the *Choosing Wisely Campaign*, Wu et al, 2020, go as far as urging a reduction in the utilization or complete elimination of the ESR from hospital test menus (44). This is due to the antiquated nature of the ESR. In the few autoimmune conditions where the ESR may provide useful information, like rheumatoid arthritis (RA), other clinical assays, such as anticyclic citrullinated peptide antibodies or rheumatoid factor, provide more specific and just as sensitive results. When combining the patient's clinical presentation with the appropriate selection of tests, the use of such a broad inflammatory biomarker is unnecessary.

#### Implementation of best practice advisory (BPA)

After the release of the *Choosing Wisely Campaign* recommendation for ESR and hs-CRP ordering*,* a handful of health systems and medical centers have implemented various versions of best practice advisories (BPA) to reduce simultaneous ordering and documented their success. Four notable publications using *Choosing Wisely Campaign* as the foundation for their quality improvement (QI) projects are reviewed here.

**Figure 5:**
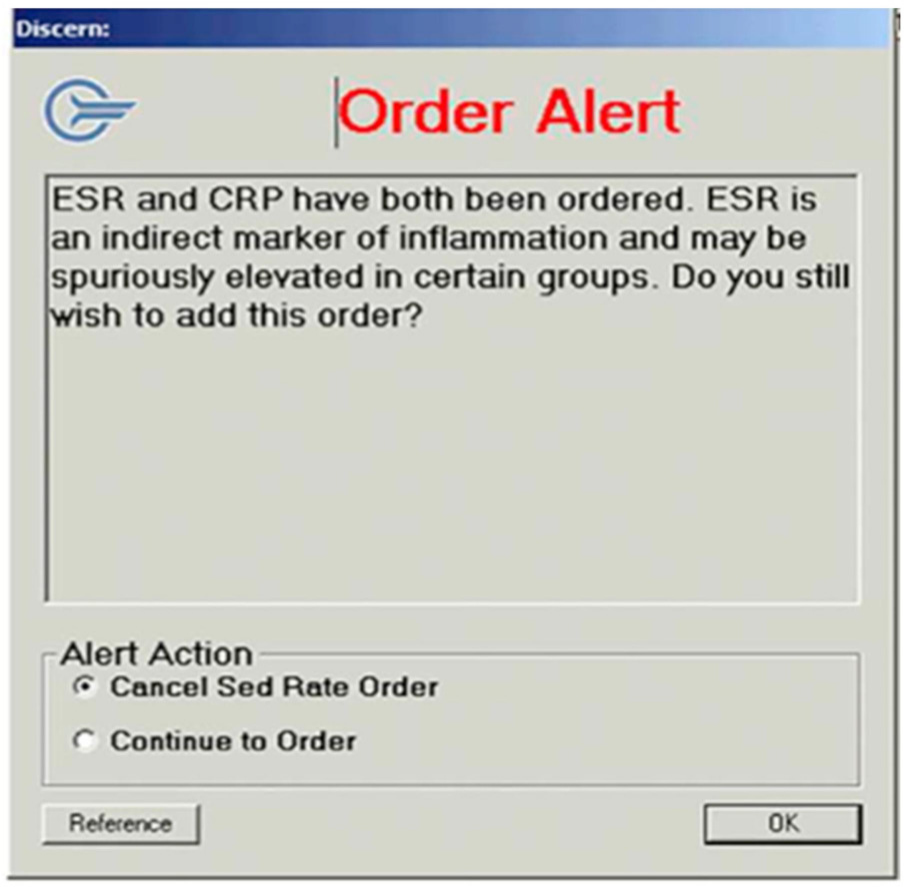
BPA - Order Alert by Juskewitch et al. (45).

Juskewitch et al. (45), utilized a computerized provider order entry decision support rule, “order alert”, to advocate against simultaneous ordering of ESR and hs-CRP from providers (Figure 5).

The authors performed a retrospective review from 2012 to 2016 recording all simultaneous orders prior to the order alert. Comparing rates before and several quarters after the CPOE implementation showed that the support rule resulted in 42% relative rate reduction in ESR/hs-CRP co-ordering and the annual payer savings of $15,000. The report projects an expected $100,000 payer cost reduction across their multi-site tertiary care setting annually and noted a substantial reduction in waste.

**Figure 6:**
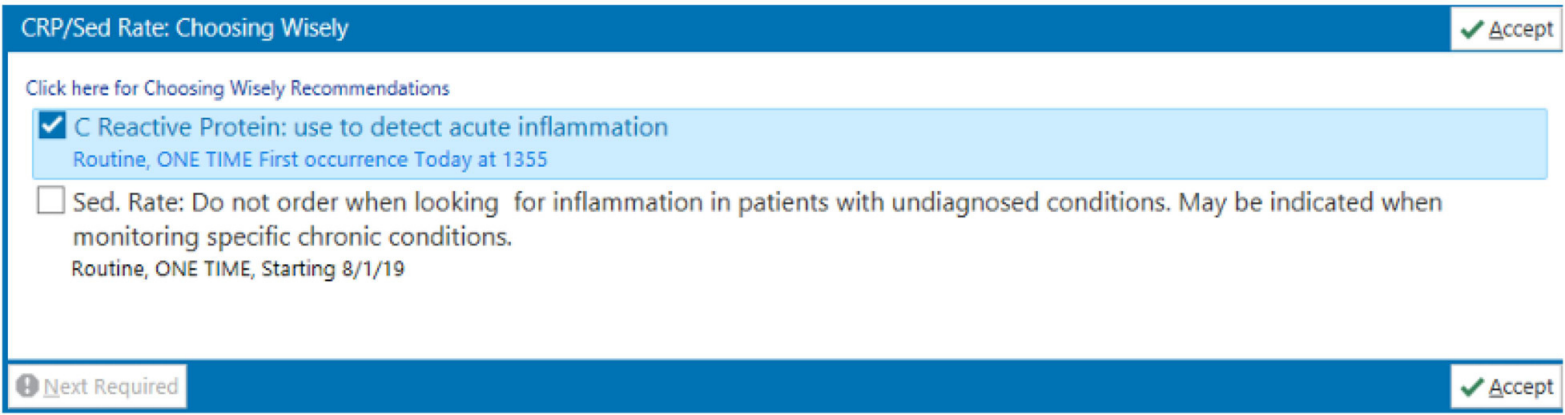
BPA - Order Alert Bartlett et al. (46).

An article using *Choosing Wisely Campaign* as the foundation for the QI project was published in 2020 from an academic medical center. Bartlett et al., utilized a survey to design interventions “focused on education, clinical decision support within the electronic medical record and quarterly audit and feedback”, noting clinical habit and ease of ordering as the reason for overuse (46) (Figure 6). To track the effectiveness of the intervention, the authors reviewed the appropriateness of ESR ordering before and after application of their intervention, as well as monthly ordering rates. These interventions achieved a 33% reduction in ESR ordering overall and a 25% reduction in ESR/hs-CRP co-ordering, which was equivalent to a reduction in 2633 ESR tests per year, with a cost savings of $23,701 annually.

**Figure 7:**
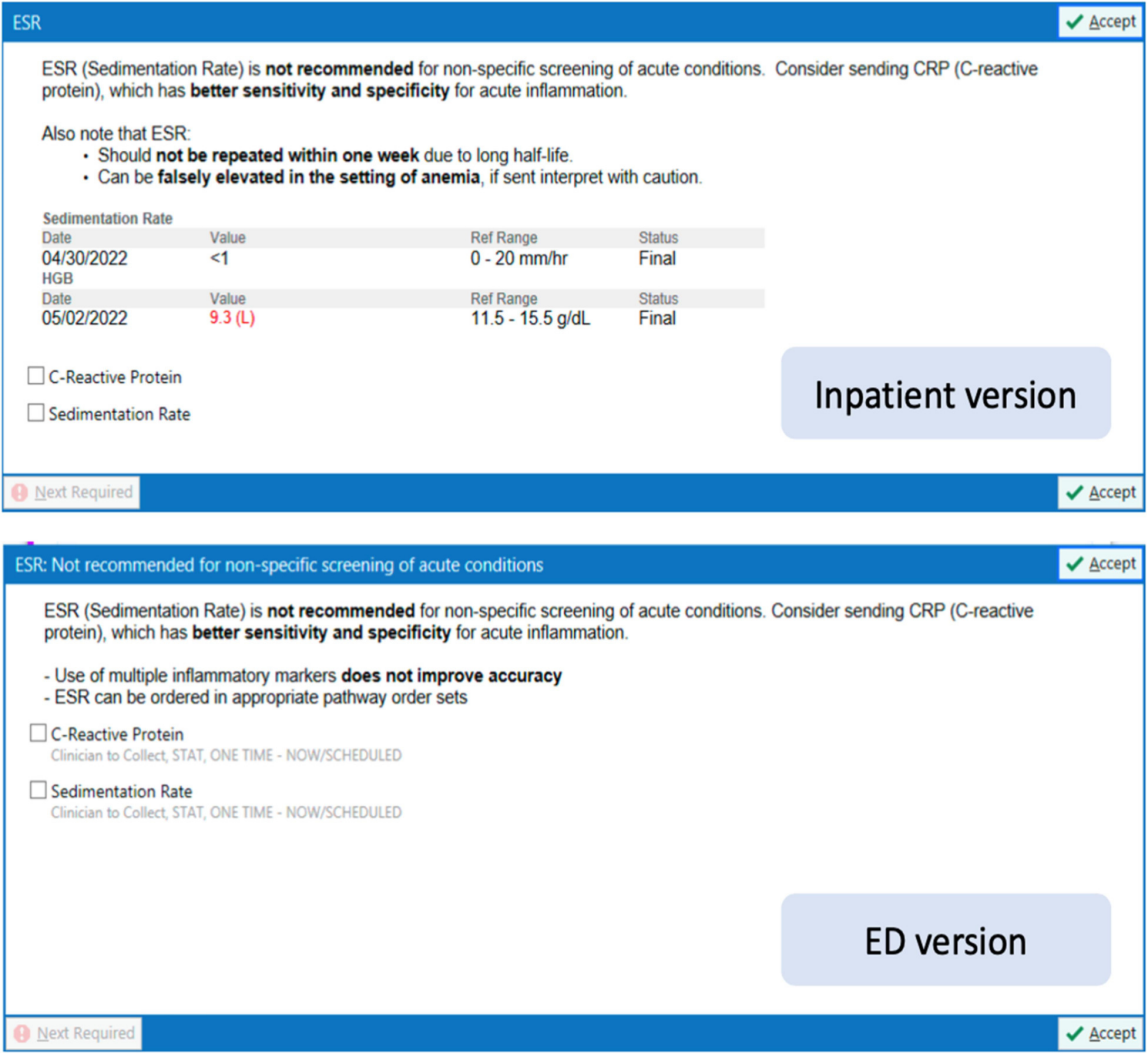
BPA - Order Alert Fatemi et al. (47).

Another documented application of the *Choosing Wisely Campaign* recommendation in the clinical setting was conducted at Children's Hospital of Philadelphia by Fatemi et al. (47) Prior to intervention at their facility, concurrent ESR and hs-CRP ordering had a rate of 80% with an unnecessary repeat order for an ESR within one week at 10%. The reason for such a high rate was thought to be due to three reasons: lack of knowledge surrounding the properties of the ESR, old habits of co-ordering both hs-CRP and ESR, and ordering with the perception that other providers would want ESR results. To mitigate the reduction of ESR ordering, two interventions were utilized. The first intervention was education and sub-specialist/ stakeholder engagement. The second intervention was applied with clinical decision support using an electronic order guidance integrated into the electronic health records (EHR), similar to the BPA used in the previously mentioned publications (Figure 7). The interventions were reviewed after one year. It resulted in a 22% decrease in inpatient ESR orders, a change from approximately 11.5 ESR tests per 100 patient days to 9 tests per 100 patient days. A 40% decrease in Emergency Department ESR orders was even more remarkable. A change from approximately 50 ESR tests per 100 patient days to 30 tests per 100 patient days. This shows that with proper intervention, geared towards both EHR modification and education of stakeholders, successful outcomes can be achieved and sustained.

**Figure 8:**
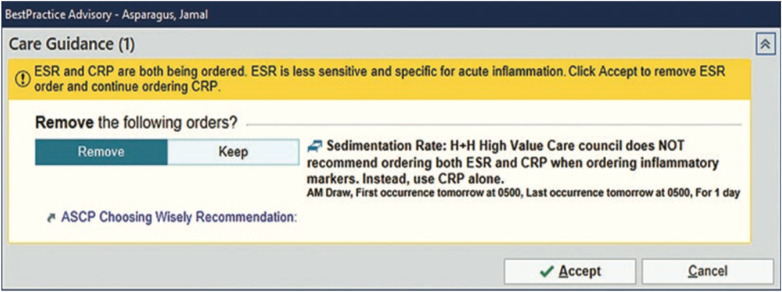
BPA - Order Alert Cho et al. (48).

Cho et al. (48), is the most recent to cite ASCP's *Choosing Wisely Campaign* as a basis for the monitoring and intervention of ESR and hs-CRP orders at their 11 hospitals and 70 ambulatory centers of a New York City health system. The authors implemented a best practice advisory (BPA) that was triggered when ESR and hs-CRP were simultaneously ordered (Figure 8). The BPA did not prevent providers from ordering both tests together, but it did provide a recommendation against that kind of practice. The BPA led to a statistically significant reduction in inpatient ESR ordering from 12.02 per 1,000 patient days before the BPA to 5.61 per 1,000 patient days after its activation (53% reduction). Similar results were achieved in their outpatient ESR orders. Outpatient ESR orders fell from 6.09 to 4.07 per 1,000 patient encounters (33% reduction). This QI project showed best success at a New York City Health System among all the published QI projects.

## Methods

The retrospective review was performed within the Veteran Affairs Northern California Healthcare System (VANCHCS), primarily at Mather VA Hospital. VANCHCS is a healthcare system comprised of one main hospital and 13 subsidiary facilities that provides various medical services and shares one laboratory information system, VISTA. The <100 bed hospital provides emergency and urgent care services, and has an intensive care unit. The study was deemed a QI project by VANCHCS. The approval of the Institutional Review Board (IRB) was not needed.

### Retrospective study on hs-CRP and ESR correlation

To apply best practice recommendations to the targeted population, VANCHCS patients within the VA healthcare system, the ESR and hs-CRP values should be correlated among the veteran patients. However, since minimal documentation was found regarding the veteran population, a correlation study was conducted. A retrospective review of all co-ordered ESRs and hs-CRPs from the past 12 months were pulled utilizing the VISTA laboratory information system (LIS). The pull included all co-ordered ESR and hs-CRP results from the past year (September 1st, 2022, to August 31st, 2023). Tests were assumed co-ordered if ordered by the same provider and drawn within six hours or less of each other. ESR and hs-CRP orders greater than six hours apart were excluded due to the rapid nature of hs-CRP secretion and clearance in response to acute inflammation and resolution. Duplicate and repeated test results were removed from the sample pool.

In addition to test values, the data included the patient’s age, the patient's gender, and order location for each test to determine patient demographics and departmental ordering frequencies. Over 30 ordering locations were listed in the data set and were categorized into eight groups based on service (see Table 1 in Results and Discussion). The categories were determined by grouping departments with similar services and ordering practices, including frequencies of co-ordering.

**Table 1:** hs-CRP and ESR co-ordered frequencies from various departments.

Designation	Departments	Frequency (n)
ED & UC	Emergency department and urgent care	1,696
PC	Primary care	1,651
Rh & Endo	Rheumatology and endocrinology	958
ID etc.	Infectious disease department, orthopedics department, podiatry, and wound care	623
Misc.	Allergy clinic, dermatology, chiropractic, dental, behavioral health, physical therapy, pain management, neurology, eye care, ENT, surgery, radiology, laboratory, GYN and pharmacy	237
Floor	Hospital floors and intensive care unit	229
Services	hem/onc, pulmonology, cardiology, vascular, hepatology, nephrology, and GI	166
Unknown	Orders without departments	284

### BPA on ESR and data collection

**Figure 9:**
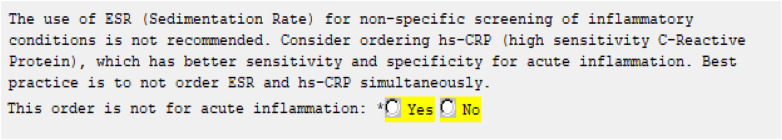
BPA - order alert utilized for this QI project.

To reduce simultaneous co-ordering of ESR and hs-CRP, two interventions were implemented. Firstly, the study used educational seminars and infographic postings to increase the awareness to physicians and practitioners in the VANCHCS on the utility and limitations of the ESR. A total of three seminars were given. The first one was given to all residents rotating through the main facility. The second talk was given to all hospitalist attendings, and the third was given at the infectious disease department. The talk consisted of an introduction to the QI project, the reason for the project, and the literature recommending a shift away from co-ordering habits. This was accomplished in a 30-minute session for each. All three educational seminars were given during the first week of November 2023. Secondly, a best practice advisory (BPA) was tied to all ESR orders placed in the Computerized Patient Record System (CPRS) (Figure 9).

The advisory was built into CPRS by the Clinical Application Coordinator (CAC) and went live on November 15th, 2023. An email was broadcasted to all physicians and practitioners two weeks prior to the go-live date. Refer to Supplementary Material for email template. Furthermore, the email content was also posted in Emergency Department resident and attending work rooms, and in all resident floor workrooms at the main facility to increase awareness of BPA implementation.

The advisory requested acknowledgment of the order as a yes or no radio button; however, it did not block provider ordering. The order was placeable regardless of the acknowledgment choice. This was intended to influence providers ordering hs-CRP away from ESR and allow co-ordering if desired.

To assess the efficacy of the interventions, the data from two months preceding the intervention (September 2023 and October 2023), one month during the intervention (November 2023) and two-month post-intervention (December 2023 and January 2024) was pulled using the same parameters of the initial 12-month retrospective review. In addition to co-ordered ESR and hs-CRP, the total number of monthly ESR or hs-CRP orders itself and monthly blood count (CBC) orders were all obtained. CBC orders are used to establish a baseline for the total test volume to rule out unaccounted variables impacting the test volume of the assays. ESR and hs-CRP ordering rates were adjusted according to the test volume represented by CBC orders for comparison and statistical analysis.

### Data analysis

For the retrospective study of hs-CRP and ESR correlation, the individual test values of ESR and hs-CRP from September 1st, 2022, to August 31st, 2023, were plotted and correlation was determined using Pearson's Correlation Coefficient, r. An r value of 0.5 or larger indicates a moderate or greater correlation. The t-test was used for statistical analysis. The *p* value is considered to be statistically significant at 0.05.

For the study of BPA and other interventions on reduction of ESR and hs-CRP concurrent orders, two t-tests were used to determine if the intervention led to a statistically significant change to ordering practices. An independent t-test was used to compare the 14 months before the intervention (12-month retrospective months and two pre-intervention months) with the post-intervention months. A paired t-test compared ordering departments two months before the intervention and two months after the intervention was used to assess departmental changes.

### Financial impact analysis

A cost-analysis was performed to assess the financial impact of the interventions. The total monthly cost for the number of ESR and hs-CRP prior to the intervention was established based on the monthly average. Monthly savings were calculated using the ESR and hs-CRP testing volume averaged from the two months post-intervention. An approximate annual and ten-year projected saving is calculated. Medical waste and technologist time are not incorporated into the cost analysis.

## Results and discussion

### Retrospective study of ESR and hs-CRP

**Figure 10:**
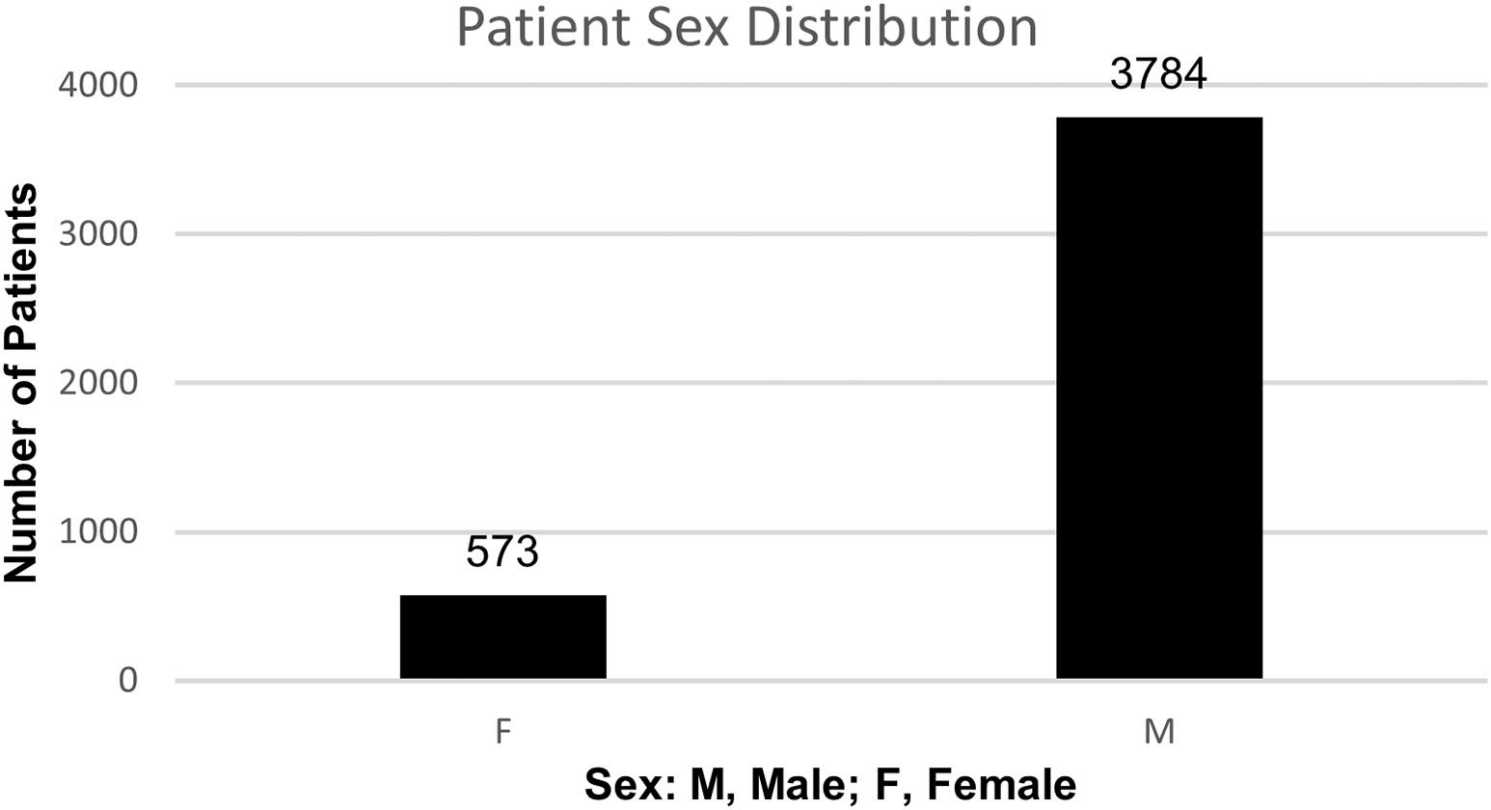
Patient Sex Distribution with ESR/hs-CRP co-orders at VANCHCS during Aug 2022 – Sept 2023.

**Figure 11:**
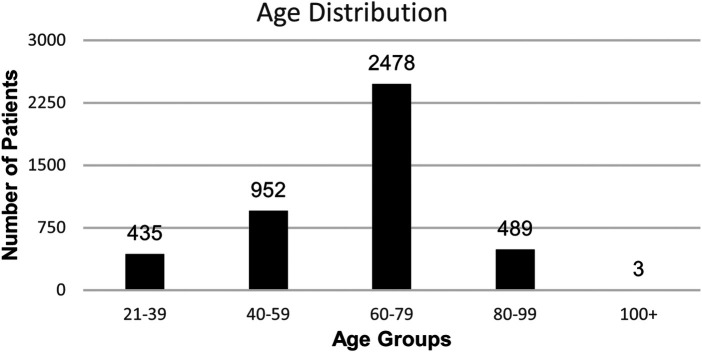
Patient Age Distribution with ESR/hs-CRP co-orders at VANCHCS during Aug 2022 – Sept 2023.

The one-year retrospective review, starting September 1st, 2022, through August 31st, 2023, included a total of 5839 paired ESR and hs-CRP orders from 4,357 different patients. All patients were veterans treated at VANCHCS. The population was comprised predominantly of male patients as expected in a VA healthcare system, 87% male (*n* = 3,784) vs. 13% female (*n* = 573) (Figure 10). For demographic analysis, ages were separated into five groups: 21 to 30 years old, 40 to 59 years old, 60 to 79 years old, 80 to 99 years old, and 100 + years old. The primary age group was 60 to 79 years old (*n* = 2,478) and accounted for 57% of all patients. The patient population's average age was 64 years old. The least frequent age group was 100 + years old with a frequency of <1% (*n* = 3) (Figure 11).

The 5,839 paired orders were categorized into eight groups depending on the origins of test orders. The top three groups were the emergency department & urgent care (ED & UC), primary care (PC), and rheumatology & endocrinology (Rh & Endo) with 1,696, 1,651, and 958 co-orders, respectively. The rest of paired orders are from the infectious disease department, orthopedics department, podiatry, and wound care (ID etc., *n* = 623), allergy clinic, dermatology, chiropractic, dental, behavioral health, physical therapy, pain management, neurology, eye care, ears nose and throat (ENT), surgery, radiology, laboratory, gynecology (GYN) and pharmacy (Misc., *n* = 237), hospital floors and the intensive care unit (Floor, *n* = 229), hem/onc, pulmonology, cardiology, vascular, hepatology, nephrology, and gastro-intestinal (GI) (Services, *n* = 166). There were 284 orders without locations that were excluded from the data. (Unknown, *n* = 284) (Table 1).

**Figure 12:**
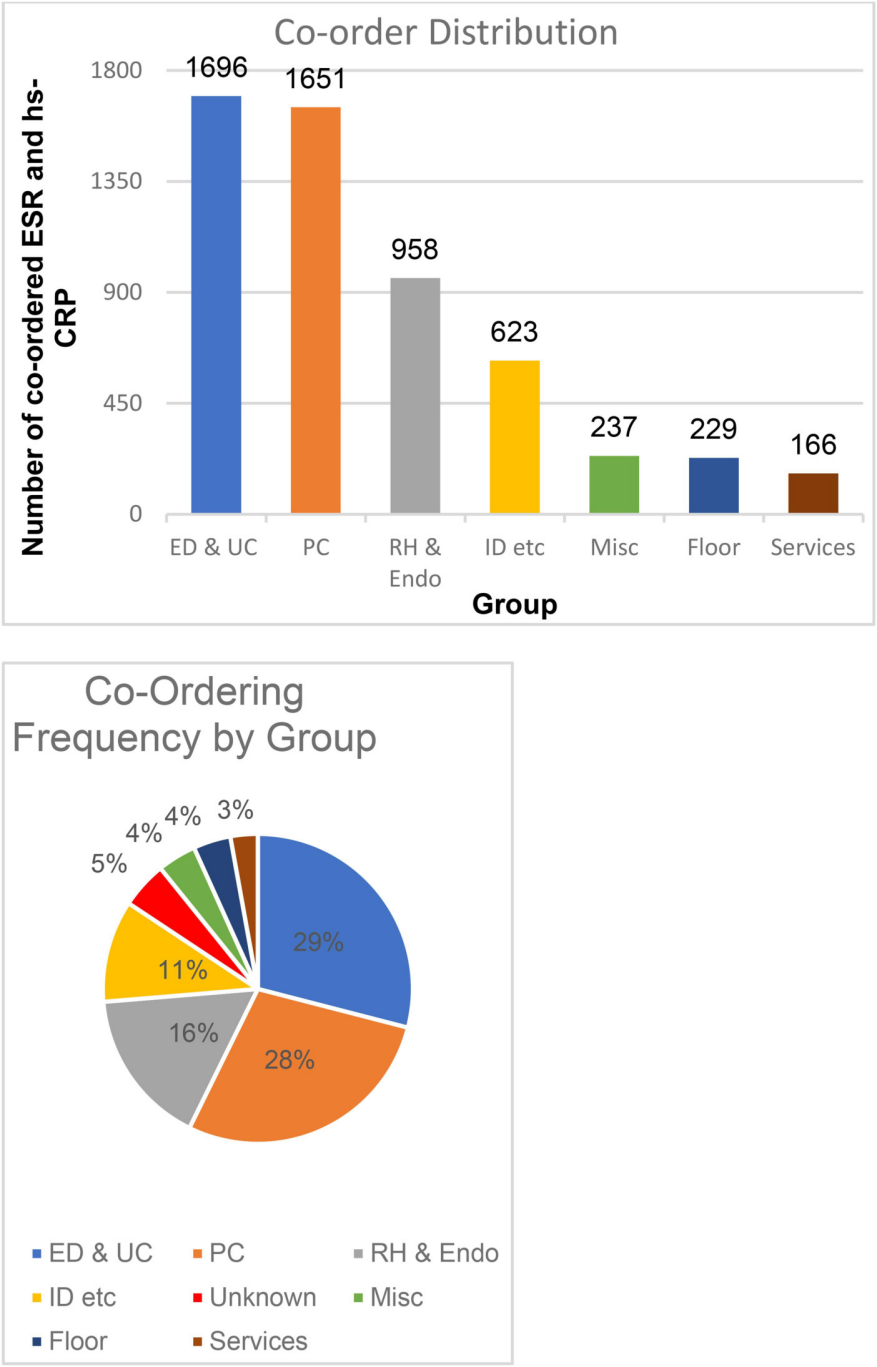
Co-ordering distribution and frequency by group.

The emergency department & urgent care, primary care, and rheumatology & endocrinology were the top ordering percentages with 29%, 28%, 16% of the annual co-orders, respectively. Together, the three groups accounted for 73% of all co-orders for the year (Figure 12).

### ESR and hs-CRP correlation

To investigate and confirm the correlation of hs-CRP and ESR test results among veterans at VANCHCS, Pearson's coefficient, r, was used to assess the association. The 5,839 paired ESR and hs-CRP results from the yearlong retrospective review had a correlation coefficient r = 0.692. This is suggestive of a moderate to strong correlation between ESR and hs-CRP. The t-value was calculated using t-score = 73.236, df = 5,837 for statistical significance, the corresponding *p*-value <0.001, indicating a significant correlation between the hs-CRP and ESR. This agrees with published literature and is the rationale behind the recommendations of the *Choosing Wisely Campaign*. The interventions by education and BPA implementation, therefore, have been conducted to reduce simultaneous testing orders of ESR and hs-CRP.

Of the 5,839 orders, 80.8% of orders correlated, either both hs-CRP and ESR being elevated or both being within normal limits, whereas 19.2% of co-orders did not agree and were discordant. The 80.8% correlation is higher than the literature reported ∼70%, likely reflecting unique patient composition and ordering practices at this VA healthcare system.

Of note here, the 19.2% were considered discordant if one of the following qualifications were met: the ESR was elevated above the upper limit of normal (greater than age divided by two in mm/hr) and the hs-CRP was below that of the upper limit of normal (< 10 mg/L), or if the hs-CRP was above the upper limit of normal and the ESR was considered normal. The former results most likely point to chronic inflammatory or other ESR-specific conditions. And it supports the continued use of ESR tests. The later discordant results support preferred hs-CRP orders alone without added diagnostic confusion from normal ESR.

### Reduction of hs-CRP and ESR co-orders by education and BPA intervention

**Figure 13:**
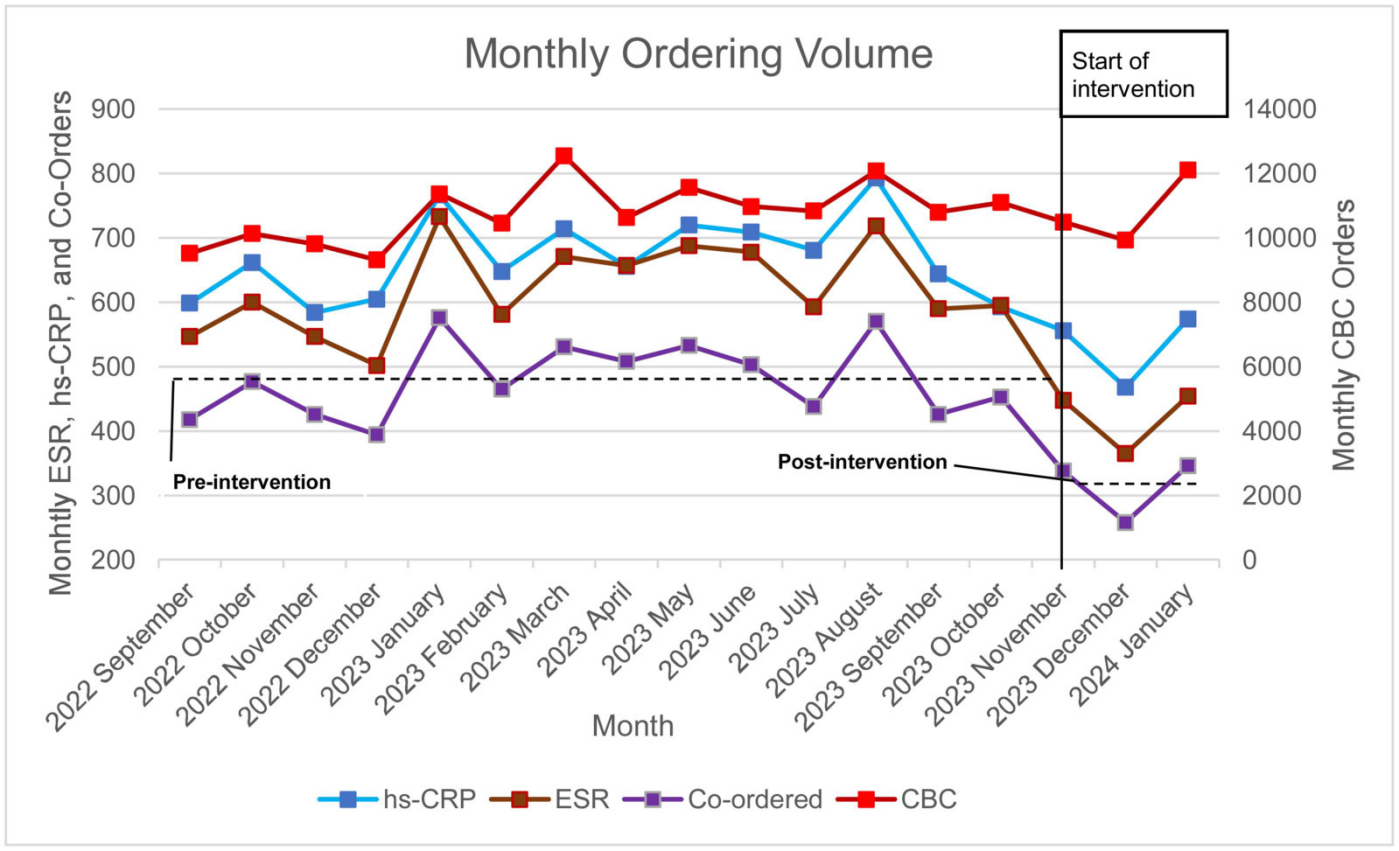
Orders Rates per Month, September 2022 to January 2024.

The monthly hs-CRP, ESR, and co-ordering frequencies from the retrospective 12-month period, including the two pre-intervention months, (total 14 months) was compared with one-month intervention and two-month post-intervention. During the fourteen months prior to intervention, there was an average of 670 total hs-CRP orders per month and 622 total ESR orders per month (Table 2). Of these, a monthly average of 480 co-orders per month was observed. The co-ordering rate, counted as the number of hs-CRPs co-ordered with ESRs out of the total number of hs-CRP orders, in the pre-intervention months was 72% (480/670). After starting the intervention, the total monthly average for hs-CRP orders fell to 533 and 422 for ESR orders (Figure 13).

**Table 2:** Comparison of monthly ordering average in pre-intervention months (September 2022 – October 2023) to intervention/post-intervention months (November 2023 – January 2024).

Tests	Pre-intervention months															Intervention	Post-Intervention			% change
	22-Sep	22-Oct	22-Nov	22-Dec	23-Jan	23-Feb	23-Mar	23-Apr	23-May	23- Jun	23- Jul	23-Aug	23-Sep	23-Oct	Ave.	23-Nov	23-Dec	24-Jan	Ave.	
hs-CRP	599	662	584	605	767	648	714	656	720	709	681	793	644	593	**669**	556	468	574	**532**	**−20%** ^a^
ESR	547	600	547	502	733	581	671	657	688	678	593	719	590	595	**621**	448	365	454	**422**	**−32%** ^b^
Co-Orders	418	477	426	394	576	465	531	508	533	503	438	570	426	453	**479**	338	258	346	**314**	**−35%** ^c^
CBC	9,526	10,142	9,812	9,316	11,372	10,460	12,549	10,635	11,567	10,979	10,836	12,079	10,801	11,106	**10,798**	10,495	9,929	12,107	**10,843**	**0.42**

^a^ p-value = 0.0042, ^b^p-value = 0.00039, ^c^p-value = 0.00036.

During the intervention and post-intervention months, co-ordering dropped to an average of 314 orders per month. This is more than a 34% decrease in co-orders per month. To assess the statistical significance in the change of co-ordering due to the intervention, a *p*-value was calculated using an independent t-test to compare 12-month retrospective review months and pre-intervention months to the intervention and post-intervention months. A t-value of 4.58 was obtained; the corresponding *p*-value = 0.00036 confirmed this was a statistically significant decrease in co-ordering. There was also a significant decrease in both hs-CRP orders (−23%, *p*-value = 0.0042) and ESR orders (35%, *p*-value = 0.00039) (Table 2). Adjusting the ordering frequency for average test volume and comparing to the number of CBC orders, the same statistical significance was obtained (Supplementary Material).

Figure 13 and Table 2 indicate that the reductions observed are due to the interventions, rather than an incidental change in test volume. The increase seen in the January post-intervention month correlates to the test volume increase seen during this month and indicates the decrease in co-ordering was sustainable over three months.

Given interventions were directed at reducing co-ordering through ESR ordering, the significant decrease seen in ESR ordering was expected. However, the interventions promoted utilizing hs-CRP, because of this an increase in hs-CRP ordering was expected. Contrary to the expectation, the overall hs-CRP order count had a significant reduction. Several unanticipated factors may have contributed to the decrease in hs-CRP utilization. The most reasonable assumption is the interventions brought awareness to ordering practices and made clinicians more vigilant and/or confident in determining when ordering an hs-CRP would add clinical benefit.

**Figure 14:**
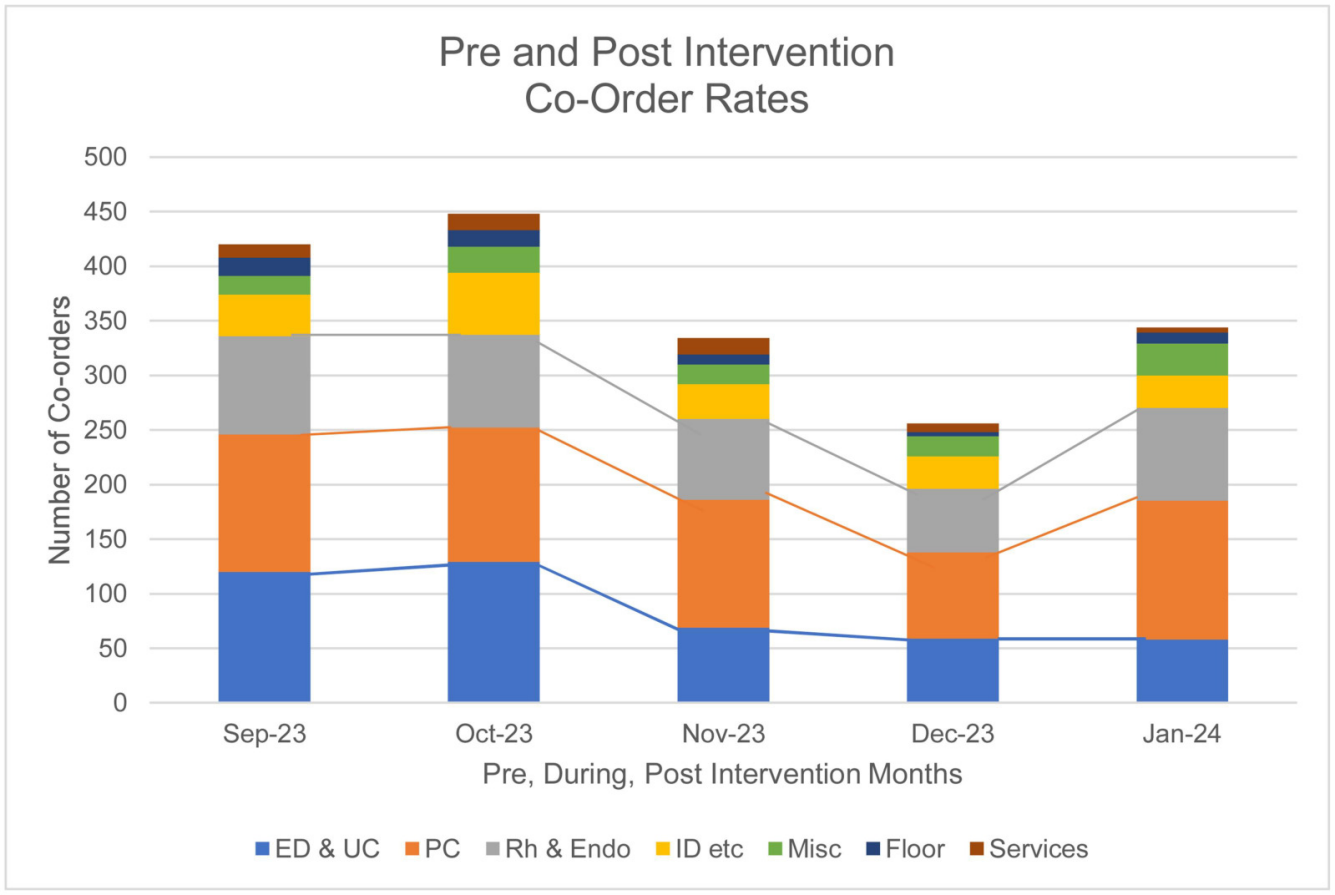
Co-Ordering Rates September 2023 to January 2024 per Group.

An assessment of the ordering departments was also conducted. Five months were used to assess the impact of the advisory and education intervention on the eight ordering groups. The ordering locations before the intervention (two months prior), during intervention (one month during the intervention), and post-intervention (two months after implementation) were categorized into the same groups as the 12-month retrospective review. The pre-intervention months, September and October, had 433 and 453 ESR/hs-CRP co-orders, respectively. The intervention month, November, had 338 co-ordered assays. The post-intervention months, December and January, had 258 and 346 co-ordered assays, respectively (Figure 14 and Table 3).

**Table 3:** Departmental Co-ordering rates Pre-intervention, intervention, and post-intervention months.

Category	Pre-intervention months			Intervention month	Post-intervention months			% Change ((Postintervention- Preintervention)/Pre intervention)x100
	Sep-23	Oct-23	Average	Nov-23	Dec-23	Jan-24	Average	
ED & UC	120	129	124.5	69	59	58	58.5	- 53.0%
PC	126	123	124.5	117	79	127	103.5	- 16.9%
Rh & Endo	90	85	87.5	74	58	85	71.5	- 16.9%
ID etc.	38	57	47.5	32	30	30	30	- 36.8%
Misc.	17	24	22.0	18	18	29	23.5	+ 6.8%
Floor	17	15	16.0	9	4	10	7.0	- 56.3%
Services	12	15	13.5	15	8	5	6.5	- 51.9%

The largest average decrease in co-ordering habits is seen in the floor with a reduction of 56.3%. The department with the lowest decrease was primary care with a reduction of 16.9%. This was expected, as primary care did not receive the educational intervention. A *p*-value for statistical significance for the decreases observed was not calculated due to the low number of post interventions month available; nevertheless, a substantial decrease is noted. The only services with an increase in co-ordering was from the misc. category, Allergy Clinic, Dermatology, Chiropractic, Dental, Behavioral Health, Physical Therapy, Pain Management, Neurology, Eye Care, ENT, Surgery, Radiology, Laboratory, Pharmacy, and GYN, with an increase of about three orders per month (Percent increase = 6.8%) (Table 3).

A small decrease in ordering was expected, as these services did not receive an educational intervention; however, the increase in ordering was unanticipated. Due to the low frequency of co-ordering, the lack of an educational intervention may have led to a clinician's ordering practice to drastically impact co-ordering rates of the misc. category as a whole.

### Comparison to literature

This QI project paralleled the previously mentioned publications. The authors, Juskewitch et al., Bartlett et al., Fatemi et al, and Cho et al., noted an overall reduction of rates in co-ordering ranging from 22% to 42%. This QI project attained a comparable decrease in co-ordering rates of 34%. In addition to overall ordering, Fatemi et al. documented a 40% drop in Emergency Department ESR orders. Implementing an order advisory and similar education, a decrease of 53% in co-orders was achieved at VANCHCS. This was the largest decrease seen among departments. Cho et al. documented the largest decrease of nearly 90% for one of their facilities. Although this decrease was not achieved in any specific locations; overall the interventions used are just as effective as reported in publications. There was a comparable significant reduction in co-ordering rates, thus this QI project is considered a success overall.

### Financial cost analysis

The cost per ESR test is approximately $1.50. Comparing this to average monthly orders from the pre-intervention months equates to $9,325 per month spent on ESR orders. The decrease in ESR orders during the two post-intervention months led to an average cost savings of $318 per month for the laboratory. The cost per hs-CRP assay is approximately $2.00 with an average total of $1,339 per month. The intervention led to a reduction in hs-CRP assay, which resulted in a cost savings of $297 per month during the post-intervention months for the laboratory. Accounting for the savings accrued from reduced ESR and hs-CRP testing, an estimated annual cost savings of $7,500 is expected, with a ten-year cost savings of $75,000. This equates to 7,458 ESRs and 8,035 hs-CRPs reduced orders per year. Further data needs to be collected in the upcoming months to determine if the savings are sustained for the remainder of the year. A cost savings analysis for medical waste reduction and technologist time was not calculated.

### Limitations

Due to limitations of the electronic health record, the diagnosis was not included in the initial data pull of 5,839 paired ESR and hs-CRP. Further investigation should be conducted to assess the impact of the patient’s condition and agreement between the ESR and hs-CRP results. The discordant results may be due to autoimmune conditions or from differing half-lives of the inflammatory markers.

The hs-CRP was expected to increase in response to the reduction in ESR ordering. The significant decrease in hs-CRP may be due to more awareness when ordering. A survey was initially discussed to determine the reason for the change but was not implemented. The study is unable to conclusively identify reasons for hs-CRP ordering reduction.

## Conclusion

The ESR and hs-CRP are cornerstone biomarkers used to assess inflammation. There is a significant correlation in the detection of inflammation between the two markers, making the co-ordering of these assays potentially wasteful. In this study, implementation of advisories and education to ordering clinicians led to a notable reduction in co-ordering and better test utilization. Further investigation should be conducted to better characterize conditions where co-ordering may be necessary. Additionally, investigation should be conducted into the ordering awareness of clinicians to narrow individual variability and build consensus in selecting laboratory assays.

## Data Availability

The original contributions presented in the study are included in the article/Supplementary Material, further inquiries can be directed to the corresponding author.
