# Supplementary material for: Reduction of simultaneous ordering rate of high sensitivity C-reactive protein and erythrocyte sedimentation rate in a veteran affairs healthcare system
Source: Front Health Serv. 2026 Apr 22;5:1729117. doi: 10.3389/frhs.2025.1729117 (PMC13144006; doi:10.3389/frhs.2025.1729117)

**Supplemental Material**

**Supplemental 1. Patient co-ordered ESR and hs-CRP values plotted for correlation**

r = 0.692

**Supplemental 2. Ordering Rates from September 2022 to January 2024, Relative to 12-month Pre-intervention Average**

Start of intervention

**Supplemental 3. Email to clinicians**


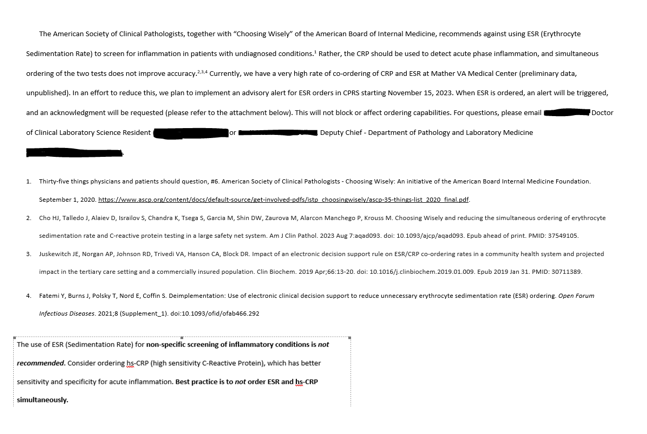

Supplement: Supplementary file 1 [file Supplementaryfile1.docx]
